# Inequalities in relative cancer survival by race, immigration status, income, and education for 22 cancer sites in Canada, a cohort study

**DOI:** 10.1002/ijc.35337

**Published:** 2025-01-16

**Authors:** Talía Malagón, Sarah Botting‐Provost, Alissa Moore, Mariam El‐Zein, Eduardo L. Franco

**Affiliations:** ^1^ Division of Cancer Epidemiology, Department of Oncology McGill University Montréal Québec Canada; ^2^ St Mary's Research Centre Montreal West Island Integrated University Health and Social Services Centre Montréal Québec Canada; ^3^ Department of Epidemiology, Biostatistics and Occupational Health McGill University Montréal Québec Canada

**Keywords:** adults, cancer survival, health inequalities, race, socioeconomic status

## Abstract

There is a paucity of disaggregated data to monitor cancer health inequalities in Canada. We used data linkage to estimate site‐specific cancer relative survival by race, immigration status, household income, and education level in Canada. We pooled the Canadian Census Health and Environment Cohorts, which are linked datasets of 5.9 million respondents of the 2006 long‐form census and 6.5 million respondents of the 2011 National Household Survey. Individual‐level respondent data from these surveys were probabilistically linked with the Canadian Cancer Registry up to 2015 and with the Canadian Vital Statistics Death database up to 2019. We used propensity score matching and Poisson models to calculate age‐standardized relative survival by equity stratifiers for all cancers combined and for 22 individual cancer sites for the period 2006–2019. There were 560,905 primary cancer cases diagnosed over follow‐up included in survival analyses; the age‐standardized period relative survival was 72.9% at 5 years post‐diagnosis. 5‐year relative survival was higher in immigrants (74.1%, 95%CI 73.8–74.4) than in Canadian‐born persons (69.6%, 95%CI 69.4–69.8), and higher in racial groups with high proportions of immigrants. There was a marked socioeconomic gradient, with 11%–12% lower relative survival in cancer patients in the lowest household income and education levels than in the highest levels. Socioeconomic gradients were observed for most cancer sites, though the magnitude varied by site. The observed differences in relative survival suggest there remain important inequities in cancer control and care delivery and quality even in a universal healthcare system.

## Graphical abstract

What's new?

While net cancer survival has markedly improved in Canada over the past decades, it is unclear whether all Canadians have benefited equally due to a paucity of data disaggregated by individual‐level equity stratifiers. Using data linkage of the census with the cancer registry, the authors provide nationally representative estimates of relative cancer survival by race, immigration status, household income, and education level that can be used to monitor health equity. Adjusting for differential background mortality using matching methods, they found important differences in cancer survival, suggesting inequities in cancer control and care delivery remain even in a universal healthcare system.

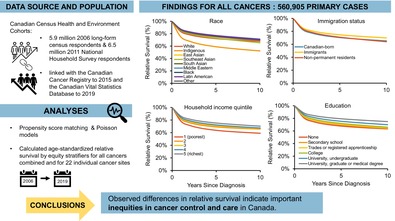

## 1 INTRODUCTION

There is a paucity of data to monitor and address cancer health inequalities in Canada.^1^ While net cancer survival has markedly improved over the past few decades,^2^ it is unclear whether all Canadians have benefited equally from these improvements. Data from other countries have documented significant inequalities in cancer survival by social equity stratifiers such as race, ethnicity, education, income, and immigration status.^3^, ^4^ Some studies have examined survival by these health equity stratifiers in Canada, but they have generally been restricted to analyses from individual provinces (mostly Ontario).^5^, ^6^, ^7^, ^8^, ^9^ It has proved more challenging to provide pan‐Canadian‐level statistics because the Canadian Cancer Registry (CCR) does not collect sociodemographic data beyond age, sex, and postal code. National‐level statistics on cancer survival inequalities have, therefore, mostly focused on location‐based inequalities such as survival by province, rurality, and ecological neighborhood‐level socioeconomic status based on residential postal code,^10^, ^11^ rather than individual‐level equity stratifiers.

Cancer survival is generally reported by cancer registries using relative survival as the estimate of net survival to cancer.^12^, ^13^ Relative survival is calculated by dividing the observed survival in cancer cases by the expected survival, generally estimated using general population life tables. Relative survival is interpretable as the expected probability of cancer survival in the absence of other causes of death. Part of the difficulty in estimating cancer survival disaggregated by health equity stratifiers comes from a lack of quality background mortality data to calculate expected survival. For example, relative survival by race in the US can be estimated due to the availability of life tables by race.^14^ Canadian life tables are only stratified by age, sex, and province.^15^ Because the background risk of non‐cancer mortality differs by socioeconomic and demographic factors, it is not possible to calculate relative survival with life tables unless sociodemographic data can be linked to both cancer registries and vital statistics databases.^12^ The validity of relative survival is also predicated on the assumption that life tables provide a good estimate of the expected mortality risk that a person with cancer would have experienced had they not had a cancer diagnosis (the counterfactual).^13^ This assumption is questionable, as cancer cases likely differ from the general population on a number of social determinants affecting both cancer and non‐cancer mortality. Methods to estimate background mortality based on more variables than those typically included in life tables could potentially lead to improved estimates of relative survival, especially when comparing groups with different background mortality rates.

Probabilistic data linkages of the CCR and vital statistics with survey data by Statistics Canada make it now possible to examine health inequalities by individual‐level stratifiers in cancer relative survival at the national level. Our primary objective was to estimate relative cancer survival in Canada stratified by race, income, education, immigration status, and cancer site in a representative sample to assess the presence of inequalities for health equity monitoring at the pan‐Canadian level. Our secondary objective was to compare two methods for estimating the expected background mortality in different groups and assess its impact on relative survival estimates: matching of cancer cases to controls versus group‐specific life tables.

## 2 METHODS

### 2.1 Data sources

The CCR collects data on all new primary cancer cases diagnosed among Canadian residents since 1992. It covers all provinces and territories; however, cancer cases from the province of Québec are missing after 2010, because at the time of the data linkages we use in this analysis the province of Québec had not submitted data to the CCR since 2011. The Canadian Vital Statistics Death (CVSD) database collects information on all deaths in Canada; deaths for Yukon are however missing since 2017 as Yukon has not yet submitted data to the CVSD since 2017. We used the CCR to identify primary cancer diagnoses and the CVSD to identify their date of death.

The 2006 Canadian long‐form census was a mandatory survey targeting a representative sample of 20% of the population residing in Canada. It collected information on the demographic, social, and economic characteristics of households to support planning and government activities. In 2011, the long form was replaced with the voluntary National Household Survey (NHS), which targeted 33% of the population and collected similar information as the census. While the voluntary NHS had a lower response rate (68.6%) than the mandatory 2006 long‐form census (93.8%), measures were deployed to offset data quality risks and validate the representativity of results using other data sources.^16^ We used the self‐reported data from these surveys and linked tax data on household income to define health equity stratifiers.

The Canadian Census Health and Environment Cohorts (CanCHECs) 2006 and 2011 are probabilistic linkages of the respondents of the 2006 long‐form census and 2011 NHS with the CCR and CVSD.^17^ The linkage rates were 90.8% and 96.7% for 2006 and 2011 respondents, corresponding to 5.9 and 6.5 million individuals, respectively. Data were linked to the CCR up to 2015, and to the CVSD up to 2019. We pooled both cohorts to increase sample size and follow‐up time; our study sample should therefore be considered to reflect the population of Canada over a period spanning 2006–2011 rather than the population at a specific point in time.

### 2.2 Health equity stratifiers

Health equity stratifiers were based on respondent data from the long‐form and NHS. Race is a social construct based on perceived differences in physical appearance, and was categorized into the following mutually exclusive groups based on Canadian Institute for Health Information recommendations^18^: Black, East Asian, Indigenous, Latin American, Middle Eastern, South Asian, Southeast Asian, White, and Other. The Other group includes those who do not identify with any of the named groups or who identify as a mix of different groups. Income was measured as the total after‐tax income from all household members, based on either linkage to tax files or self‐report in the census questionnaire. Household income was adjusted by an equivalency factor which accounts for household size, and categorized into quintiles at the national level. Education level was measured according to a person's highest completed degree. A Canadian‐born person is someone born in Canada and a citizen by birth. An immigrant is a person who has been granted the right to live permanently in Canada by immigration authorities, including both naturalized citizens and permanent residents. A non‐permanent resident is a person who has a temporary work/study permit or is a refugee claimant.

Prior to data release, the long‐form and NHS data underwent editing and imputation operations by Statistics Canada to address inconsistent and missing responses to the questionnaires, including deterministic and nearest‐neighbor imputation. The imputation rates for the questions related to these health equity stratifiers were of the order of 1.3%–5.5% depending on the question and on census year.

### 2.3 Cancer case inclusions

We used a period survival analysis approach; the period approach uses the conditional survival probabilities of cancer cases to derive the most up‐to‐date estimates of expected cancer survival and is the preferred method for estimating survival in population‐based cancer registries.^19^ We included both incident cases diagnosed during follow‐up (2006–2019) and cases who were within 10 years of their diagnosis on census day (mid‐May 2006 or 2011). We included primary cancers among individuals aged 15 to 99 years at diagnosis; this age range was selected for consistency with national cancer statistics.^20^ We excluded cases whose diagnosis was established by autopsy only or death certificate only, cases with a missing diagnosis date, and cases whose death date preceded their diagnosis date. We allowed multiple primary cancers per person to contribute to analyses if they were diagnosed in different years. We excluded cancer cases from CanCHEC 2011 which were already included in CanCHEC 2006 to avoid duplicate observations. Cases were classified by cancer site using the grouping definitions of the 2021 Canadian Cancer Statistics (Supplementary Table 3).^20^

Follow‐up started either on the date of cancer diagnosis for incident cases, or on census day for cases diagnosed prior to the census day. The period approach of estimating survival accounts for the left‐truncation of data and avoids potential immortal‐time bias from cases diagnosed prior to start of follow‐up by excluding person‐time at risk prior to the start of follow‐up and using only conditional survival probabilities by time since diagnosis on the date of start of follow‐up.^19^ For example, a case diagnosed with cancer 3 years prior to the census day would only start contributing time at‐risk in analyses after year 3 and therefore only contributes to 4–10 years survival estimates. We however excluded cancer cases diagnosed prior to census day specifically for the analyses stratified by income to prevent reverse causality, as a previous cancer diagnosis can potentially affect a household's income. Cases contributed person‐time to interval‐specific conditional survival analyses for each year they were alive during follow‐up, up to 10 years after their cancer diagnosis or the last date of data linkage on December 31, 2019, except cases from Yukon for which follow‐up ended on December 31, 2016.

## 3 STATISTICAL ANALYSES

### 3.1 Relative survival based on matched controls

In our main analysis, we estimated relative survival using matching of cancer cases to controls to estimate the expected background mortality risk. Controls were selected among CanCHEC respondents who had no prior record of a site‐specific cancer diagnosis in the CCR since 1992, and who were alive during the year of the diagnosis of their matched cancer case. Controls were matched to cancer cases using propensity score matching, using greedy nearest neighbor matching without replacement.^21^ The propensity score models included age, sex, race, household after‐tax income quintile, education level, immigration status, neighborhood income level quintile, province of residence, rurality of residence, and calendar year as predictors of cancer diagnosis. Exact matches were requested for age, sex, race, and calendar year. Separate propensity score models were fit for each cancer site. Up to three controls were selected for each cancer case, except for the model for all cancer sites combined, where only 1 control was selected per case to reduce computational burden. The index date of start of follow‐up for controls was the date of start of follow‐up of their matched cancer case. We excluded controls whose date of death preceded the index date. Estimates were weighted using propensity matching weights.

We calculated relative cancer survival for each year up to 10 years post cancer diagnosis using a Poisson regression model. Dickman et al. (2004) proposed that relative survival could be estimated assuming a Poisson process for the number of deaths by interval since diagnosis, which assumes a piecewise constant mortality hazard function.^22^ We modified the parameterization of their proposed model to fit a Poisson model estimating the number of deaths from all causes ($\mu_{i}$) for observation *i*:

$$\ln\left(\mu_{i}\right)=\beta_{0}+{\mathrm{age}}_{i}\beta_{1}+{\mathrm{sex}}_{i}\beta_{2}+{\text{interval}}_{i}\beta_{3}+{\text{cancer}}_{i}\beta_{4}+{\text{interval}}_{i}*{\text{cancer}}_{i}\beta_{5}+{\text{stratifier}}_{i}\beta_{6}+{\text{stratifier}}_{i}*{\text{cancer}}_{i}\beta_{7}+\ln\left({\mathrm{PY}}_{i}\right),$$

where ${\mathrm{age}}_{i}$ is a categorical variable for age at diagnosis (15–44, 45–54, 55–64, 65–74, and 75–99 years), ${\mathrm{sex}}_{i}$ is a categorical variable for sex, ${\text{interval}}_{i}$ is a categorical variable indicating the time since cancer diagnosis in 1‐year intervals, ${\text{cancer}}_{i}$ indicates whether *i* is a cancer case or a control, ${\text{stratifier}}_{i}$ is a health equity stratifier of interest, and ${\mathrm{PY}}_{i}$ are person‐years at risk during the follow‐up interval.

This model estimates the excess mortality rate in cancer cases compared to controls by time interval since diagnosis (parameters $\beta_{4}$, $\beta_{5}$), and allows different groups to have different excess cancer mortality rates ($\beta_{7}$) while controlling for differences in background mortality from other causes ($\beta_{6}$). We used a type 3 test of parameters to assess whether the excess cancer mortality rate differs across groups. The model parameters were used to estimate mortality rate ratios using the exponent of model parameters, and the cumulative survival by time *t* ($S_{t,i}$) in cases and controls using the transformation of the hazard approach, where ${X_{i}}^{\prime }\beta$ is the vector of model covariates:

$$S_{t,i}=e^{\left(-\sum_{\text{interval}=1}^{t}\left(e^{X_{i}\prime \beta }\right)\right)}$$

Cancer relative survival (${\mathrm{RS}}_{t,i}$) was then calculated by dividing the expected survival for cancer cases by the expected survival for controls of the same age, sex, and stratifier group. Confidence intervals (95%CI) for relative survival were calculated using the log–log transformation and the delta method with the R *msm* package.^23^, ^24^

### 3.2 Relative survival based on life tables

In secondary analyses, we recalculated relative survival using group‐specific life tables. The objective of this secondary analysis was to obtain relative survival estimates comparable to those reported by the Canadian Cancer Statistics for validation of sample representativity, and to compare results with relative survival estimated with the aforementioned matching methods. We calculated age‐ and sex‐specific life tables for the entire CanCHEC 2006 and 2011 cohorts by race, immigration status, income quintile, and education level. We used the hazard transformation approach to estimate relative cancer survival using the period method.^25^ Estimates were weighted using CanCHEC survey weights. CIs for relative survival estimates were obtained through bootstrapping, using the 2.5th to 97.5th percentiles of 500 CanCHEC‐specific bootstrapping weights. The Supplementary Material provides more details on the methods used to generate life tables, relative survival estimates, and background mortality rates (Supplementary Figure 1).

### 3.3 Age standardization

Relative survival estimates were age‐standardized using the Canadian Cancer Survival Standard age weights for individual cancer sites.^2^, ^20^ For all cancers combined, we used the international single standard of the EUROCARE‐2 study.^26^ For Poisson models, we specified age weights in estimating equations to estimate age‐standardized survival. For survival calculated with life tables, we used direct standardization.

### 3.4 Validation of estimates

To assess whether our survival estimates are representative of the Canadian population, we compared the relative survival estimates based on life tables in CanCHECs with official statistics of relative cancer survival based on life tables for all of Canada. Because our survival estimates span the period from 2006 to 2019, we compared our results to national relative survival estimates for 2006–2008 and 2015–2017 to define the target range.^20^, ^27^

### 3.5 Protection of confidentiality

Analysis outputs were vetted using rules developed by Statistics Canada, which include rounding all counts to base 5 and not disclosing statistics for groups with less than 5 contributing events.

## 4 RESULTS

### 4.1 Baseline characteristics of cancer cases and controls

The demographic characteristics of the CanCHECs have previously been described.^17^, ^28^ There were 562,905 cancer cases and 560,665 matched controls eligible for all cancer sites combined period survival analyses (Table 1). Cancer cases had a mean age at diagnosis of 63.3 years (standard deviation 14.0), and 50% were female. The majority self‐identified as White (88%) and Canadian‐born (75%). Cases and matched controls were comparable in terms of age, sex, race, immigration status, province of residence, rurality, household income, neighborhood income, and education level.

**TABLE 1:** Baseline characteristics of cancer cases aged 15–99 years at diagnosis and matched controls for all sites combined, CanCHECs 2006 & 2011.

	Cancer cases	Matched controls
Number^a^	562,905	560,665
Mean age at diagnosis (SD)^b^	63.3 (14.0)	63.4 (14.0)
	**(%)**	**(%)**
Sex		
Female	49.8	49.8
Male	50.2	50.2
Age group on July 1 of matching year^c^		
<15 years^c^	<0.1	<0.1
15–19 years	0.2	0.2
20–24 years	0.5	0.5
25–29 years	0.8	0.8
30–34 years	1.2	1.2
35–39 years	1.8	1.8
40–44 years	3.0	3.0
45–49 years	5.2	5.2
50–54 years	8.0	8.0
55–59 years	10.9	10.9
60–64 years	13.6	13.6
65–69 years	14.3	14.3
70–74 years	13.5	13.5
75–79 years	11.9	11.9
80–84 years	8.7	8.7
85–89 years	4.7	4.8
90+ years	1.8	1.8
Racial group		
White	87.7	87.7
Indigenous	3.3	3.3
East Asian	3.2	3.2
Southeast Asian	1.2	1.2
South Asian	1.9	1.9
Middle Eastern	0.6	0.6
Black	1.3	1.3
Latin American	0.4	0.4
Other	0.4	0.4
Immigration status		
Canadian‐born	74.8	74.8
Immigrants	25.0	25.0
Non‐permanent residents	0.1	0.2
Province/territory		
Newfoundland and Labrador	1.7	1.9
Prince Edward Island	0.4	0.5
Nova Scotia	3.4	3.7
New Brunswick	2.7	2.9
Québec	15.2	15.1
Ontario	44.1	43.2
Manitoba	4.0	4.2
Saskatchewan	3.3	3.5
Alberta	10.0	10.1
British Columbia	14.9	14.6
Yukon	0.1	0.1
Northwest Territories	0.2	0.2
Nunavut	0.1	0.1
Residence area		
Rural	21.5	22.0
Urban	78.5	78.0
Household after‐tax income quintile, adjusted for household size		
1 (poorest)	17.2	17.2
2	21.7	21.6
3	20.4	20.4
4	19.7	19.8
5 (richest)	21.0	21.0
Neighborhood income quintile^d^		
1 (poorest)	18.3	18.2
2	19.6	19.7
3	20.1	20.1
4	20.3	20.4
5 (richest)	21.7	21.7
Education level^e^		
None	28.2	27.5
Secondary school	23.4	23.0
Trades or registered apprenticeship	12.2	12.3
College	15.6	16.0
University, undergraduate degree	15.8	16.1
University, graduate or medical degree	4.8	5.0
Missing (<15 years on census day^c^)	0.1	0.1

Abbreviation: SD, standard deviation.

^a^ Number of cases rounded to base 5 for confidentiality.

^b^ For controls, age on the matched cancer diagnosis date.

^c^ Cases and controls were matched on age on July 1 of the year of cancer diagnosis (incident cases) or the census year (cases diagnosed prior to census day). Some cases and controls aged 14 on census day or July 1 were eligible if the cancer diagnosis date occurred in the second half of the year when they were 15 or older. Education information was not collected for individuals who were <15 years old on census day in mid‐May.

^d^ Neighborhood income quintile was derived using residential postal code with the Postal Code Conversion File Plus (PCCF+) based on 2006 census data.

^e^ The following responses were included in each category. None: No certificate, diploma or degree. Secondary school: High school diploma or equivalent. Trades or registered apprenticeship: Registered Apprenticeship certificate; Other trades certificate or diploma. College: College, CEGEP (junior college) or other non‐university certificate or diploma from a program of 3 months to less than 1 year; a program of 1 year to 2 years; or a program of more than 2 years. University undergraduate degree: University certificate or diploma below bachelor level; Bachelor's degree; University certificate or diploma above bachelor level. University, graduate or medical degree: Degree in medicine, dentistry, veterinary medicine or optometry; Master's degree; Earned doctorate.

### 4.2 Relative survival compared to matched controls

Poisson model predictions for relative cancer survival by time since diagnosis for the overall cohort of cancer cases are presented in Table 2. Mortality rates for cancer cases were highest in the year following diagnosis; the mortality rate for all cancers combined was 16.4 times higher (95%CI 15.9–16.8) in cancer cases than in controls the first year after diagnosis, and declined to 1.4 times higher (95%CI 1.4–1.5) in cancer cases than in controls by the 10th year after diagnosis. The predicted age‐standardized relative survival for all cancers combined was 84.0% 1 year post‐diagnosis, 72.9% 5 years post‐diagnosis, and 68.4% 10 years post‐diagnosis.

**TABLE 2:** Mortality rate ratios and cumulative relative survival at 1, 5, and 10 years after cancer diagnosis, ages 15–99 years at diagnosis, estimated using two different methods.

			Mortality rate ratio (cancer cases vs. controls)			Relative survival compared to controls (%)^b^			Relative survival compared to life tables (%)^b^		
Cancer site	Cancer cases^a^ (*n*)	Controls^a^ (*n*)	1 year	5 years	10 years	1 year	5 years	10 years	1 year	5 years	10 years
All cancers	562,905	560,665	16.4 (15.9–16.8)	2.1 (2.0–2.1)	1.4 (1.4–1.5)	84.0 (83.9–84.2)	72.9 (72.7–73.1)	68.4 (68.2–68.6)	78.6 (78.5–78.8)	64.5 (64.4–64.7)	58.8 (58.6–59.0)
Head and neck	19,975	59,575	8.6 (7.5–9.8)	2.4 (2.1–2.7)	2.2 (1.8–2.5)	88.0 (87.3–88.6)	72.7 (71.6–73.7)	63.5 (62.2–64.9)	83.0 (82.3–83.8)	63.0 (61.8–64.1)	52.2 (51.1–53.5)
Esophagus	4690	13,910	28.4 (23.7–34.1)	4.1 (3.1–5.4)	1.6 (1.0–2.5)	46.5 (44.5–48.6)	18.1 (16.4–19.8)	14.8 (13.2–16.6)	42.2 (40.2–44.1)	15.5 (14.0–16.8)	11.7 (10.4–13.1)
Stomach	9345	27,700	22.9 (20.1–26.2)	2.5 (2.1–3)	1.6 (1.2–2.1)	52.6 (51.3–54.0)	30.7 (29.3–32.0)	27.1 (25.7–28.6)	47.3 (45.9–48.7)	26.2 (24.8–27.5)	22.6 (21.2–23.9)
Colorectal	84,010	250,260	7.5 (7.1–8.0)	1.9 (1.8–2.0)	1.3 (1.2–1.4)	86.0 (85.7–86.4)	70.6 (70.1–71.2)	65.5 (64.8–66.1)	82.3 (81.9–82.8)	64.6 (64.1–65.2)	58.2 (57.4–58.9)
Liver	4530	13,430	32.7 (26.9–39.9)	4.9 (3.7–6.4)	2.5 (1.5–4.0)	46.0 (43.9–48.0)	21.9 (20.1–23.7)	14.7 (13.1–16.4)	41.9 (39.7–44.1)	19.7 (18.3–21.0)	13.1 (11.7–14.4)
Pancreas	10,215	30,260	50.2 (44.7–56.5)	3.8 (3.0–4.7)	2.1 (1.5–3.1)	24.3 (23.4–25.2)	7.0 (6.4–7.5)	5.7 (5.1–6.3)	22.7 (21.6–23.7)	7.1 (6.5–7.6)	5.6 (5.1–6.2)
Lung and bronchus	61,820	183,640	32.9 (31.2–34.8)	3.5 (3.3–3.8)	2.2 (2.0–2.5)	44.4 (44.0–44.9)	21.4 (21.0–21.8)	15.9 (15.5–16.3)	40.6 (40.1–41.2)	18.3 (17.9–18.7)	12.8 (12.4–13.1)
Melanoma	29,255	87,340	3.2 (2.8–3.7)	1.7 (1.5–2.0)	1.0 (0.9–1.2)	97.7 (97.4–97.9)	92.7 (92.1–93.2)	90.5 (89.6–91.3)	95.6 (95.1–96.1)	87.2 (86.3–88.0)	83.0 (81.7–84.1)
Breast (female)	116,375	348,200	3.1 (2.9–3.4)	2.2 (2.0–2.3)	1.6 (1.5–1.8)	98.0 (97.8–98.1)	91.6 (91.3–91.9)	86.3 (85.8–86.7)	97.1 (96.9–97.3)	88.6 (88.3–89.0)	82.2 (81.7–82.8)
Cervix (female)	7410	22,190	16.3 (10.7–24.7)	4.7 (3.1–7.1)	1.6 (1.0–2.4)	91.7 (90.7–92.6)	78.6 (77.0–80.1)	75.3 (73.5–77.0)	88.8 (87.6–90.1)	73.5 (72.0–75.1)	69.6 (67.8–71.3)
Uterus (female)	25,285	75,635	6.5 (5.4–7.6)	1.9 (1.6–2.2)	1.2 (1.0–1.4)	93.6 (93.1–94.1)	83.5 (82.6–84.3)	81.0 (79.8–82.1)	91.5 (90.8–92.2)	79.9 (78.7–81.0)	77.3 (75.8–78.7)
Ovary (female)	9745	29,110	16.9 (13.8–20.7)	6.6 (5.4–8.1)	2.6 (1.9–3.6)	80.2 (79.1–81.2)	48.0 (46.6–49.4)	38.4 (37.0–39.9)	76.8 (75.5–78.2)	45.3 (44.0–46.7)	36.8 (35.4–38.0)
Prostate (male)	111,480	332,860	2.1 (2.0–2.2)	1.2 (1.2–1.3)	1.1 (1.0–1.2)	98.2 (98.1–98.4)	95.1 (94.7–95.4)	92.8 (92.2–93.3)	97.1 (96.9–97.4)	92.3 (91.8–92.6)	88.4 (87.8–89.0)
Testis (male)	5540	16,620	6.5 (2.9–14.7)	0.6 (0.2–2.0)	1.3 (0.5–3.2)	98.9 (98.4–99.3)	98.0 (97.2–98.6)	97.4 (96.4–98.1)	98.5 (97.9–98.9)	96.8 (95.9–97.5)	96.0 (94.9–96.9)
Bladder	33,810	100,545	4.5 (4.1–4.9)	1.6 (1.5–1.8)	1.3 (1.2–1.5)	91.6 (91.1–92.0)	81.4 (80.6–82.2)	74.9 (73.7–76.1)	88.2 (87.6–88.8)	74.1 (73.2–75.0)	64.3 (63.0–65.5)
Kidney and renal pelvis	20,795	62,070	10.0 (8.8–11.4)	1.9 (1.7–2.2)	1.6 (1.3–1.9)	87.6 (86.9–88.2)	78.0 (77.1–79.0)	71.9 (70.6–73.1)	83.3 (82.5–84.2)	70.5 (69.4–71.6)	62.7 (61.4–63.9)
Brain & CNS	7370	22,000	55.2 (44.7–68.1)	7.8 (6.1–10.2)	3.6 (2.5–5.2)	51.0 (49.4–52.5)	18.8 (17.6–20.1)	13.5 (12.4–14.6)	47.7 (46.3–49.0)	22.7 (21.7–23.7)	18.0 (17.0–19.1)
Thyroid	23,270	69,685	4.1 (3.1–5.3)	1.3 (1.0–1.7)	0.8 (0.6–1.0)	99.3 (99.1–99.4)	98.9 (98.6–99.1)	98.6 (98.2–98.9)	98.3 (98.0–98.6)	97.2 (96.8–97.7)	96.2 (95.4–96.7)
Hodgkin Lymphoma	5105	15,285	14.6 (8.5–25.0)	2.6 (1.6–4.4)	2.4 (1.4–4.2)	96.1 (95.3–96.8)	92.4 (91.1–93.5)	89.4 (87.7–90.9)	91.4 (89.9–92.8)	84.6 (82.7–86.3)	78.8 (77.0–80.9)
Non‐Hodgkin Lymphoma	29,760	88,690	10.6 (9.6–11.7)	2.0 (1.8–2.3)	1.7 (1.5–2.0)	84.8 (84.3–85.4)	74.8 (74.0–75.6)	66.6 (65.5–67.6)	79.3 (78.6–80.0)	66.3 (65.4–67.2)	57.2 (56.1–58.2)
Multiple myeloma	7620	22,660	11.3 (9.6–13.3)	5.1 (4.3–6.1)	4.0 (3.1–5.2)	78.9 (77.7–80.0)	49.6 (48.0–51.2)	27.8 (26.1–29.5)	75.5 (74.1–76.8)	44.9 (43.3–46.3)	26.5 (24.8–28.2)
Leukemia	17,540	52,220	12.6 (11.2–14.1)	2.3 (2.0–2.6)	2.0 (1.6–2.3)	78.0 (77.1–78.8)	65.4 (64.2–66.5)	56.4 (55.0–57.8)	71.2 (70.2–72.2)	56.8 (55.6–57.9)	47.4 (46.2–48.6)

*Note*: Numbers in parentheses are the 95% confidence interval.

Abbreviation: CNS, central nervous system.

^a^ Counts are rounded to base 5. The all cancers model includes a different number of cancers than the sum of individual cancer sites due to the restriction to one primary cancer per year per person for the all cancers analysis, and due to the inclusion of other rarer cancer sites. Controls were matched to cases at a ratio of up to 1:1 (all cancers) or 3:1 (individual cancer sites).

^b^ Age‐standardized using the Canadian Cancer Survival Standard weights (individual cancer sites) or the international single standard (all cancers combined).

**FIGURE 1:**
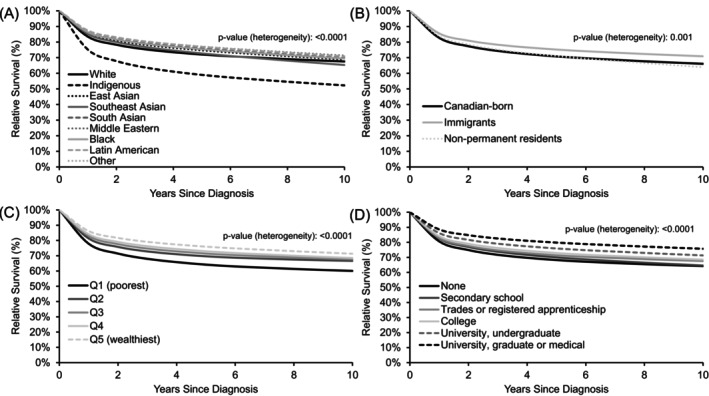
10‐year relative survival for all cancers combined compared to matched controls, ages 15–99, stratified by (A) race, (B) immigration status, (C) household income quintile, and (D) education level. *P*‐values represent a type III test for the interaction effect of each characteristic on the excess cancer mortality rate. Estimates are age‐standardized using the weights of the international single standard from the EUROCARE‐2 study.^26^

There were significant differences in relative survival by race, immigration status, household income, and education level for all cancers combined (Figure 1, Tables 3 and 4). Specifically, the 5‐year relative cancer survival was lower in White (70.9%, 95%CI 70.7–71.1) and Indigenous (57.3%, 95%CI 57.9–59.4) persons than in other racial groups (71.0–75.8%), and was higher in immigrants (74.1%, 95%CI 73.8–74.4) than in Canadian‐born persons (69.6%, 95%CI 69.4–69.8) (Table 3). There was a strong gradient in relative cancer survival by household income and education level, with 5‐year relative cancer survival being lowest in the poorest income quintile (63.0%, 95%CI 62.5–63.5) and highest in the richest income quintile (74.8%, 95%CI 74.4–75.2), and lowest in those with no diploma (67.1%, 95%CI 66.8–67.4) compared with those with a graduate or medical university degree (78.8%, 95%CI 78.2–79.4) (Table 4). Similar social gradients were observed across most individual cancer sites, though the type 3 test for heterogeneity was not always significant due to low case numbers for some cancer sites, leading to higher uncertainty in estimates. Estimates for all 10 years of follow‐up by group can be found in the Supplementary Table 1.

**TABLE 3:** Age‐standardized 5‐year relative survival (%) by cancer site, race, and immigration status, ages 15–99 years at diagnosis, compared with matched controls.

	Racial group										Immigration status			
Cancer site	White	Indigenous	East Asian	Southeast Asian	South Asian	Middle Eastern	Black	Latin American	Other	*p*‐value^a^	Canadian‐born	Immigrants	Non‐permanent residents	*p*‐value^a^
All cancers	70.9 (70.7–71.1)	57.3 (56.4–58.2)	73.3 (72.5–74.0)	71.0 (69.7–72.4)	74.5 (73.4–75.5)	75.8 (73.9–77.5)	74.0 (72.7–75.2)	75.6 (73.2–77.8)	73.9 (71.3–76.2)	<.0001	69.6 (69.4–69.8)	74.1 (73.8–74.4)	69.4 (65.7–72.8)	.0009
Head and neck	69.7 (68.6–70.8)	54.9 (49.8–59.7)	70.8 (66.1–75.0)	71.8 (62.3–79.4)	64.1 (58.2–69.3)	77.3 (63.2–86.5)	67.0 (55.3–76.2)	76.6 (58.0–87.7)	69.7 (54.1–80.9)	<.0001	68.2 (67.0–69.4)	72.5 (70.7–74.3)	63.4 (41.4–79.0)	.4035
Esophagus	16.1 (14.7–17.5)	14.3 (9.4–20.2)	15.0 (8.2–23.7)	11.3 (1.1–35.3)	26.5 (17.1–36.9)	28.1 (7.0–54.5)	8.9 (2.5–20.4)	10.1 (0.6–35.8)	28.0 (6.1–56.0)	.6141	15.3 (13.9–16.8)	19.5 (17.0–22.3)	14.0 (1.1–42.2)	.875
Stomach	27.0 (25.7–28.3)	16.2 (12.5–20.2)	40.2 (35.4–44.8)	37.6 (28.0–47.3)	30.9 (23.1–39.0)	42.9 (30.3–55)	37.4 (28.3–46.5)	33.2 (21.6–45.2)	32.9 (18.7–47.9)	.0104	24.8 (23.4–26.2)	34.0 (32.0–35.9)	22.0 (7.8–40.7)	.0687
Colorectal	68.1 (67.5–68.7)	55.8 (53.2–58.3)	71.8 (69.5–73.9)	66.3 (61.7–70.5)	72.4 (68.1–76.2)	72.9 (66.2–78.5)	64.9 (60.1–69.2)	70.7 (61.7–78.0)	71.2 (61.9–78.6)	<.0001	66.6 (65.9–67.2)	71.6 (70.7–72.5)	65.3 (52.3–75.6)	.8982
Liver	16.8 (15.3–18.4)	8.1 (5.5–11.3)	34.2 (29.9–38.5)	29.4 (21.9–37.2)	23.3 (16.0–31.3)	28.7 (13.7–45.7)	16.2 (8.5–26.0)	35.6 (17.3–54.5)	33.2 (18.3–48.9)	.4464	14.2 (12.8–15.7)	28.0 (25.6–30.5)	12.8 (0.8–41.3)	.5438
Pancreas	5.9 (5.5–6.5)	1.5 (0.9–2.4)	11.4 (8.5–14.8)	15.5 (9.6–22.7)	10.9 (7.0–15.7)	10.3 (4.1–20.1)	9.2 (5.6–13.8)	17.6 (8.1–30.1)	8.3 (2.7–18.0)	.0421	5.4 (5.0–6.0)	8.5 (7.5–9.5)	15.7 (1.6–43.6)	.7495
Lung and bronchus	17.8 (17.4–18.2)	12.6 (11.3–13.9)	26.5 (24.3–28.7)	25.6 (21.7–29.6)	22.6 (18.6–26.9)	25.4 (18.8–32.4)	18.0 (14.1–22.3)	29.3 (20.1–39.0)	28.6 (19.4–38.6)	.0001	16.7 (16.3–17.1)	22.9 (22.1–23.7)	31.1 (20.1–42.7)	.0025
Melanoma	91.3 (90.7–92.0)	81.9 (72.4–88.4)	84.1 (70.0–91.9)	82.7 (58.0–93.6)	70.9 (53.6–82.7)	96.6 (50–99.8)	89.3 (60.0–97.5)	77.2 (53.1–89.9)	94.2 (0.0–100.0)	.0876	91.3 (90.6–92.0)	90.9 (89.7–92.0)	82.8 (43.3–95.8)	.5045
Breast (female)	90.6 (90.3–90.9)	87.2 (85.4–88.8)	91.6 (90.4–92.8)	89.1 (87.0–90.8)	89.6 (87.9–91.1)	89.9 (86.8–92.4)	86.0 (83.4–88.2)	87.3 (82.6–90.7)	88.4 (84.0–91.7)	<.0001	90.3 (89.9–90.6)	90.9 (90.4–91.4)	89.5 (82.2–93.9)	.0005
Cervix (female)	77.9 (76.1–79.6)	67.9 (62.0–73.1)	86.7 (80.9–90.8)	77.7 (68.8–84.3)	84.1 (76.2–89.5)	69.3 (32.2–88.8)	81.9 (71.7–88.7)	92.1 (75.5–97.7)	90.7 (74.8–96.8)	.9473	76.2 (74.3–77.9)	83.1 (80.4–85.4)	85.2 (53.0–96.1)	.5014
Uterus (female)	83.6 (82.7–84.6)	73.8 (66.7–79.6)	81.4 (76.9–85.0)	72.8 (66.1–78.4)	78.8 (73.8–82.9)	73.9 (58.6–84.2)	66.6 (59.0–73.1)	80.9 (66.9–89.4)	72.0 (56.2–83.0)	<.0001	83.1 (82.0–84.1)	81.7 (80.1–83.1)	88.5 (62.8–96.8)	.0005
Ovary (female)	44.4 (43–45.9)	38.4 (31.6–45.2)	55.1 (48.2–61.5)	59.9 (48.9–69.3)	43.0 (36.2–49.6)	39.5 (24.9–53.6)	46.7 (34.6–58.0)	49.1 (30.4–65.3)	38.0 (21.9–53.9)	.0044	43.8 (42.2–45.4)	47.7 (45.3–50.0)	48.6 (24.9–68.7)	.3734
Prostate (male)	94.6 (94.2–94.9)	90.4 (87.5–92.7)	97.3 (95.6–98.3)	95.9 (92.2–97.9)	94.8 (92.8–96.3)	96.4 (90.5–98.6)	95.1 (92.9–96.6)	97.9 (86.3–99.7)	100.0 (0.0–100.0)	.3333	94.4 (93.9–94.8)	95.2 (94.6–95.6)	94.6 (66.3–99.3)	.3893
Testis (male)^b^	NE	NE	NE	NE	NE	NE	NE	NE	NE	NE	97.9 (97.0–98.5)	98.1 (96.6–99.0)	96.7 (78.9–99.5)	.7925
Bladder	79.4 (78.5–80.3)	65.5 (58.3–71.9)	83.9 (78.9–87.7)	68.1 (54.9–78.1)	81.7 (74.7–86.9)	81.8 (72.3–88.3)	80.8 (69.1–88.4)	86.3 (61.6–95.7)	77.7 (53.2–90.4)	.1004	78.4 (77.4–79.4)	81.8 (80.3–83.1)	77.5 (40.7–93.0)	.6235
Kidney and renal pelvis	75.8 (74.7–76.8)	67.2 (63.2–70.9)	77.6 (72.6–81.7)	70.2 (60.6–77.9)	85.1 (78.9–89.6)	86.3 (75.8–92.4)	81.1 (72.7–87.1)	76.3 (65.3–84.3)	77.1 (58.8–88.0)	.0687	74.2 (73.1–75.3)	79.7 (78.1–81.2)	89.4 (37.8–98.7)	.6081
Brain and CNS	15.9 (14.8–17.0)	19.4 (13.3–26.4)	21.6 (15.1–28.8)	25.1 (14.2–37.7)	27.6 (21.1–34.6)	16.1 (7.3–28.0)	17.0 (9.7–26.0)	25.8 (9.0–46.6)	13.1 (3.7–28.7)	.1162	15.6 (14.4–16.7)	19.8 (17.8–22.0)	13.3 (2.1–35.1)	.5642
Thyroid	98.5 (98.2–98.8)	95.6 (91.7–97.6)	99.1 (97.9–99.6)	99.0 (96.8–99.7)	98.6 (97.1–99.4)	99.2 (95.2–99.9)	99.4 (95.7–99.9)	99.0 (90.2–99.9)	98.8 (93.5–99.8)	.9853	98.4 (98.0–98.8)	98.9 (98.5–99.2)	98.9 (79.9–99.9)	.9681
Hodgkin lymphoma	90.9 (89.4–92.2)	87.2 (76.1–93.4)	92.9 (82.2–97.3)	97.1 (57.9–99.8)	92.9 (87.4–96.0)	91.7 (79.7–96.8)	93.8 (76.4–98.5)	93.4 (63.6–99.0)	87.9 (59.6–96.8)	.8883	90.9 (89.4–92.2)	92.1 (89.8–93.8)	74.4 (37.2–91.5)	.6447
Non‐Hodgkin lymphoma	72.3 (71.4–73.2)	58.5 (53.5–63.2)	71.5 (67.5–75.2)	59.0 (51.7–65.6)	65.0 (60.0–69.6)	75.5 (66.5–82.4)	59.0 (52.0–65.3)	80.6 (67.4–88.9)	71.7 (53.7–83.7)	<.0001	71.2 (70.3–72.2)	72.4 (71.0–73.8)	76.4 (48.0–90.6)	.0711
Multiple myeloma	41.0 (39.4–42.6)	29.5 (23.0–36.2)	47.4 (38.1–56.1)	60.6 (47.4–71.6)	50.8 (42.6–58.4)	43.0 (29.3–56.0)	54.3 (45.9–61.9)	55.8 (31.3–74.6)	64.4 (42.1–79.9)	.2545	40.2 (38.4–42.0)	45.7 (43.1–48.2)	66.9 (24.5–89.1)	.4162
Leukemia	63.3 (62.1–64.5)	42.1 (35.3–48.7)	46.9 (40.2–53.4)	46.5 (35.0–57.2)	65.2 (58.7–70.9)	54.1 (42.9–64.0)	47.9 (38.3–56.9)	61.4 (44.0–74.8)	67.0 (44.2–82.2)	<.0001	61.6 (60.3–62.9)	64.2 (62.3–66.1)	60.9 (39.7–76.6)	.1969

*Note*: Numbers in parentheses are the 95% confidence interval. Age‐standardized using the Canadian Cancer Survival Standard weights (individual cancer sites) or the international single standard (all cancers combined).

Abbreviations: CNS, central nervous system; NE, not estimable.

^a^ Type III test for interaction effect between the characteristic and cancer status; tests whether there is evidence of heterogeneity in excess cancer mortality rates across groups over the entire 10‐year period, not just at 5 years.

^b^ The model for testicular cancer by race did not converge due to data sparsity, with too few deaths in some racial groups to estimate relative cancer survival.

**TABLE 4:** Age‐standardized 5‐year relative survival by cancer site, household income quintile, and education level, ages 15–99 years at diagnosis, compared with matched controls.

	Household income quintile						Education level						
Cancer site	1 (poorest)	2	3	4	5 (richest)	*p*‐value^a^	None	Secondary school	Trades or registered apprenticeship	College	University, undergraduate	University, graduate or medical	*p*‐value^a^
All cancers	63.0 (62.5–63.5)	68.7 (68.3–69.1)	70.4 (70.0–70.8)	71.8 (71.4–72.2)	74.8 (74.4–75.2)	<.0001	67.1 (66.8–67.4)	68.6 (68.3–69.0)	69.6 (69.2–70.0)	71.2 (70.8–71.6)	74.8 (74.4–75.2)	78.8 (78.2–79.4)	<.0001
Head and neck	57.9 (55.2–60.5)	66.8 (64.3–69.2)	72.6 (70.1–74.9)	71.6 (69.1–73.9)	75.0 (72.7–77.1)	.0055	67.6 (65.9–69.3)	67.2 (65.3–69.1)	68.1 (65.7–70.3)	69.2 (66.8–71.5)	72.2 (69.8–74.5)	78.3 (74.2–81.8)	<.0001
Esophagus	11.3 (9.0–13.9)	18.6 (15.9–21.5)	18.0 (15.2–21.1)	15.7 (13.2–18.4)	19.2 (16.4–22.3)	<.0001	16.0 (14–18.2)	14.5 (12.4–16.7)	16.1 (13.4–19.1)	17.5 (14.7–20.6)	16.7 (13.6–20.1)	23.8 (17.3–30.9)	<.0001
Stomach	26.1 (23.5–28.9)	29.1 (26.6–31.7)	28.9 (26.3–31.6)	26.0 (23.4–28.7)	27.9 (25.3–30.7)	.0055	28.1 (26.2–30.0)	25.4 (23.2–27.7)	29.4 (26.5–32.3)	26.2 (23.4–29.1)	30.2 (27.2–33.4)	34.8 (29.3–40.4)	<.0001
Colorectal	61.6 (60.1–63.1)	67.8 (66.5–69.0)	68.0 (66.8–69.3)	69.1 (67.8–70.3)	69.4 (68.1–70.6)	<.0001	67.4 (66.5–68.3)	66.1 (65.1–67.1)	67.3 (65.9–68.6)	67.5 (66.2–68.7)	68.4 (67.1–69.6)	71.6 (69.4–73.7)	<.0001
Liver	15.8 (13.3–18.5)	18.6 (15.8–21.6)	21.8 (18.8–25.0)	18.9 (16.0–22.0)	22.5 (19.1–26.1)	.0007	17.0 (15.0–19.2)	20.4 (17.8–23.1)	18.0 (15.1–21.2)	17.5 (14.4–20.8)	23.7 (20.0–27.6)	31.8 (24.5–39.3)	.0032
Pancreas	4.7 (3.9–5.6)	5.9 (5.0–6.9)	6.5 (5.6–7.6)	7.6 (6.5–8.7)	7.5 (6.4–8.6)	<.0001	5.6 (4.9–6.4)	5.1 (4.4–5.9)	6.8 (5.7–8.0)	6.5 (5.5–7.7)	8.2 (6.9–9.5)	6.8 (5.1–8.9)	<.0001
Lung and bronchus	13.8 (13.1–14.5)	18.3 (17.6–19.0)	18.9 (18.1–19.7)	19.1 (18.2–20.0)	19.7 (18.7–20.6)	<.0001	17.0 (16.5–17.5)	17.1 (16.5–17.8)	17.9 (17–18.7)	18.1 (17.2–19.0)	21.8 (20.7–23.0)	25.6 (23.0–28.2)	<.0001
Melanoma	88.5 (85.8–90.8)	91.7 (89.9–93.2)	91.2 (89.5–92.5)	91.7 (90.2–92.9)	91.6 (90.4–92.7)	.0099	89.8 (88.4–91.1)	90.0 (88.7–91.1)	91.9 (90.2–93.3)	91.1 (89.7–92.3)	91.2 (90.0–92.3)	93.1 (91.2–94.7)	.0039
Breast (female)	88.5 (87.5–89.4)	90.6 (89.8–91.4)	91.1 (90.2–91.8)	91.4 (90.6–92.1)	91.2 (90.5–91.9)	<.0001	90.7 (90.1–91.2)	89.9 (89.4–90.5)	88.9 (87.8–89.8)	89.6 (89.0–90.2)	90.0 (89.4–90.6)	91.3 (90.1–92.4)	<.0001
Cervix (female)	74.9 (71.1–78.3)	76.5 (72.7–79.8)	75.9 (71.7–79.5)	76.6 (72.5–80.2)	80.7 (76.8–84.0)	.045	75.2 (72.2–78.0)	75.0 (72.0–77.7)	78.5 (73.5–82.7)	79.3 (76.1–82.2)	78.9 (75.4–82.0)	84.0 (75.2–89.8)	.0011
Uterus (female)	79.1 (76.4–81.6)	81.9 (79.6–84.0)	83.7 (81.5–85.6)	83.6 (81.4–85.5)	81.6 (79.5–83.4)	<.0001	81.5 (79.7–83.1)	83.1 (81.5–84.6)	83.1 (80.0–85.7)	82.7 (80.9–84.4)	82.5 (80.6–84.2)	81.7 (77.7–85.0)	<.0001
Ovary (female)	39.4 (35.9–42.9)	45.0 (41.7–48.3)	42.6 (39.5–45.8)	49.0 (45.7–52.3)	46.3 (43.2–49.4)	.0002	42.6 (40.0–45.1)	44.0 (41.6–46.4)	46.4 (41.7–51.0)	46.4 (43.6–49.3)	44.3 (41.3–47.3)	45.9 (40.2–51.5)	<.0001
Prostate (male)	92.5 (91.0–93.7)	93.4 (92.4–94.2)	94.4 (93.5–95.3)	95.2 (94.3–96.0)	95.5 (94.7–96.2)	.8367	93.2 (92.5–93.8)	94.2 (93.4–94.9)	94.9 (94.1–95.6)	95.1 (94.2–95.9)	95.5 (94.8–96.2)	94.6 (93.6–95.4)	.0051
Testis (male)	93.1 (89.3–95.6)	98.1 (94.6–99.3)	98.1 (95.4–99.2)	97.8 (95.7–98.8)	98.4 (96.7–99.3)	.6651	97.4 (95.3–98.6)	97.7 (96.1–98.6)	98.1 (95.9–99.1)	96.9 (95.0–98.1)	98.4 (97.0–99.2)	99.6 (90.5–100.0)	.439
Bladder	74.3 (71.5–76.8)	78.9 (76.8–80.8)	80.8 (78.7–82.6)	79.4 (77.3–81.3)	81.5 (79.4–83.5)	.0233	77.9 (76.5–79.3)	78.4 (76.7–80.0)	80.0 (78.0–81.8)	78.8 (76.6–80.7)	80.5 (78.4–82.3)	84.2 (81.0–87.0)	.0066
Kidney and renal pelvis	69.2 (66.4–71.7)	73.5 (71.2–75.6)	76.6 (74.4–78.7)	75.7 (73.5–77.8)	77.4 (75.2–79.4)	.0045	73.5 (71.9–75.1)	74.8 (72.9–76.6)	75.8 (73.5–78.0)	75.3 (73.1–77.4)	76.9 (74.8–78.9)	81.7 (78.0–84.9)	<.0001
Brain and CNS	16.0 (13.5–18.7)	16.4 (14.1–18.9)	17.4 (15.1–19.8)	14.6 (12.5–16.8)	15.9 (13.8–18.0)	<.0001	16.8 (14.8–18.8)	15.1 (13.3–17.0)	14.0 (11.7–16.6)	15.8 (13.7–18.0)	18.4 (16.1–20.8)	20.2 (16.2–24.6)	<.0001
Thyroid	98.0 (96.9–98.7)	98.2 (97.3–98.8)	98.6 (97.8–99.1)	98.8 (98.0–99.3)	98.8 (98.1–99.2)	.9622	98.4 (97.7–98.9)	98.2 (97.5–98.6)	98.0 (96.9–98.7)	98.7 (98.0–99.2)	98.9 (98.3–99.3)	99.1 (97.8–99.6)	.73
Hodgkin lymphoma	86.9 (82.2–90.4)	90.6 (87.2–93.2)	90.0 (86.8–92.5)	90.1 (86.9–92.5)	94.6 (92.1–96.3)	.373	90.1 (87.7–92.1)	88.6 (85.8–90.8)	90.7 (87.4–93.2)	91.3 (88.4–93.5)	93.2 (90.9–94.9)	94.4 (89.5–97.0)	.567
Non‐Hodgkin lymphoma	64.7 (62.3–67.1)	71.0 (69.1–72.9)	71.0 (69.0–72.9)	70.4 (68.5–72.3)	76.7 (74.9–78.4)	.0004	67.8 (66.3–69.3)	71.1 (69.5–72.7)	71.0 (68.9–73.0)	71.1 (69.2–73.0)	73.9 (72.0–75.6)	80.0 (77.1–82.6)	<.0001
Multiple myeloma	38.0 (33.9–42.1)	39.3 (35.9–42.7)	42.8 (39.2–46.3)	44.4 (40.8–47.9)	46.8 (43.3–50.2)	.0204	38.9 (36.4–41.4)	39.2 (36.3–42.1)	42.2 (38.4–46.0)	42.3 (38.8–45.8)	45.8 (42.4–49.2)	52.3 (46.3–58.0)	.0019
Leukemia	55.9 (52.7–58.9)	59.3 (56.8–61.7)	62.5 (59.9–64.9)	62.6 (60.0–65.1)	66.6 (64.0–69.0)	.064	59.9 (58.0–61.8)	60.6 (58.4–62.7)	61.3 (58.5–64.0)	63.4 (60.8–65.9)	63.9 (61.2–66.3)	69.5 (65.4–73.2)	<.0001

*Note*: Numbers in parentheses are the 95% confidence interval. Age‐standardized using the Canadian Cancer Survival Standard weights (individual cancer sites) or the international single standard (all cancers combined).

Abbreviation: CNS, central nervous system.

^a^ Type III test for interaction effect between the characteristic and cancer status; tests whether there is evidence of heterogeneity in excess cancer mortality rates across groups over the entire 10‐year period, not just at 5 years.

### 4.3 Relative survival compared to life tables

Relative survivals compared to life tables are presented in Table 2 for the overall population, and in Supplementary Table 2 by race, immigration status, income, and education level. Many estimates could not be reported when using life tables due to the number of cancer cases and deaths being below the confidentiality disclosure threshold of five contributing cases. The relative survival estimates using matched controls were higher than the relative survival estimates using life tables for all cancer sites, with the single exception of brain and central nervous system cancers, which had higher 5‐ and 10‐year relative survival estimates using life table methods (Table 2). While the point estimates changed using different methods, the social gradients in survival by race and income were similar between methods.

### 4.4 Validation of estimates with historical data

The relative survival estimates using life table methods in CanCHECs 2006 and 2011 were comparable to relative cancer survival statistics based on life table methods for all of Canada during the period of 2006–2017 (Supplementary Figures 2–4).

## 5 DISCUSSION

We found significant differences in relative cancer survival by race, immigration status, household income, and education level in Canada for many major cancer sites from 2006 to 2019. Relative cancer survival was worse in persons with lower household income and lower education levels. Conversely, relative cancer survival was higher in immigrants and in racial groups with high proportions of immigrants. Our validation exercise found that relative survival estimates in CanCHECs are comparable to those reported for all of Canada during the same time period when using comparable methods, and consequently, our estimates are likely representative of the Canadian population. The cancer survival differences we observed between social groups can be contextualized in terms of the historical progress in cancer control. Relative cancer survival improved by 6% over the 20‐year period spanning from 1992/1994 to 2012/2014 in Canada.^2^ The 3%–12% survival differences we observed between groups for many cancer sites are therefore analogous to being 10–40 years “behind” in benefiting from improvements in cancer control compared to more advantaged groups.

The matching process we used was intended to adjust for differences in the sociodemographic profiles between cancer cases and non‐cancer cases in order to derive valid estimates of net cancer survival within each demographic group. The matching did not however adjust for differences between demographic groups; demographic groups may differ in terms of comorbidities, health access, or health behavior differences which may be driving the differences in survival we observed. The objective of our study was descriptive, to describe true differences that exist between populations in cancer survival regardless of their underlying cause. Our objective was not to decompose the relative contribution of each health equity stratifier in determining cancer survival, as we consider the monitoring of health inequalities within each social dimension equally important. For example, it is possible that some of the differences that exist between different racial groups could be mediated by differences in education and income; this would however not change the fact that there exist fundamental differences in cancer survival between racial groups. In the case of survival combining all cancer sites together, it is also important to keep in mind that the incidence rates of different cancer types differ by race and socioeconomic status,^28^, ^29^ and that therefore some of the differences in overall cancer survival are attributable to differences in the distribution of types of cancers experienced by each demographic group. We do not consider this to be confounding, as this reflects a true difference in the aggregated survival experiences of different demographic groups when summing across all cancers.

An important limitation of our analysis is that, despite the combination of two CanCHEC cycles over multiple years of follow‐up to increase sample size, there were low event numbers for some cancer sites and demographic groups. This led to issues of power, data confidentiality, and limited ability to examine interactions between the different dimensions of health equity stratifiers affecting survival (intersectionality). While some of the differences in excess cancer mortality rates between groups were not statistically significant, this probably reflects the low number of events in some groups leading to higher uncertainty in estimates. A lack of statistical significance should not be interpreted as indicating an absence of inequalities, but rather a lower power to detect significant differences for some rarer cancers. This issue was particularly important for survival disaggregated by race, as the vast majority of cancer cases were in White persons (88%), due in part to the White population being older and having higher cancer incidence rates than other racial groups in Canada.^28^ This led to lower power to assess clinically meaningful differences in survival by race. While the effects of sex, immigration, household income, and education are also likely to differ by racial group, we were unable to assess these interactions due to low case numbers.

We found that higher household income and education level were consistently associated with improved relative cancer survival across most sites. The majority of previous studies of disparities in cancer survival in Canada have looked at neighborhood‐level rather than individual‐level measures of income.^7^, ^8^, ^9^ Our results confirm that these socioeconomic gradients apply at the individual level as well. Canada has a universal health care system funded through taxes, with all citizens and permanent residents having insurance covering most primary and hospital care.^30^ While this partly mitigates inequalities in healthcare access by income, the financial burden of a cancer diagnosis remains unequally distributed, as coverage for pharmacy‐dispensed prescription drugs and home care is more variable, and cancer patients still face indirect costs such as travel or income loss.^31^ Inequalities in health persist because individuals with more resources such as higher income and education are better able to avail themselves of preventive and therapeutic care to improve their health.^32^ Cancer screening rates display a socioeconomic gradient despite being covered by insurance in Canada.^33^, ^34^ While screening and early diagnosis may contribute to some of the observed differences, previous studies have found that adjusting for stage at diagnosis, however, only partly explains the survival gradient by socioeconomic status.^7^ Differences in treatments received may also contribute to observed differences in survival by socioeconomic status.^8^, ^9^

Studies of immigrants in Canada have documented barriers in access to healthcare such as language and cultural norms.^35^ Immigrants have lower cancer screening participation rates than Canadian‐born persons and are less likely to have their cancer detected by screening.^36^, ^37^, ^38^ Nonetheless, our study as well as others consistently find strong evidence of a healthy immigrant effect, where immigrants have generally better cancer outcomes and mortality rates than those born in Canada.^5^, ^28^, ^39^, ^40^ The majority of immigrants to Canada are economic immigrants who are selected based on their ability to contribute to Canada's economy,^41^ and are required to undergo a medical examination as part of the selection process. This selection process leads to an immigrant population with better average health and performance on a number of health indicators such as fewer chronic conditions as well as lower rates of obesity and smoking than those born in Canada.^42^ The presence of more underlying comorbidities in Canadian‐born persons could influence cancer survival through more advanced stages at diagnosis and longer diagnostic intervals, and potentially also differences in tumor biology.^43^ Like most studies of cancer survival, we are unable to account for emigration in analyses, so it is possible that our results are subject to immortal time bias due to emigration. This bias is likely stronger for immigrants than those born in Canada, as studies have found that emigration rates are higher for recent immigrants.^44^ However, we are unaware of any study of emigration rates in cancer patients specifically. We hypothesize that emigration rates would likely be lower in cancer cases than controls, as cancer patients would require active and ongoing maintenance care in the years following their diagnosis, making emigration more difficult. Therefore, emigration would likely bias estimates toward lower relative survival estimates in groups with higher emigration, as survival would be overestimated in controls (denominator) relative to cancer cases (numerator). We consequently do not believe that higher emigration rates can plausibly explain the higher relative survival in immigrants, as the direction of the bias would be expected to lead to lower relative survival estimates.

We observed large differences in relative survival by race, though these were not always significant. A large proportion of the individuals from racial groups other than White and Indigenous are immigrants,^28^ which may in part explain the higher relative cancer survival across multiple cancer sites compared with White and Indigenous cancer patients, who are predominantly born in Canada. The observed lower cancer survival in Indigenous peoples reflects the enduring legacy of colonialism, which continues to cause barriers to accessing high‐quality cancer care for the Indigenous peoples of Canada (the First Nations, Inuit, and Métis peoples).^45^ Several prior studies have examined the cancer survival experiences of different Indigenous peoples using a distinctions‐based approach.^46^, ^47^, ^48^, ^49^, ^50^

The choice of method to calculate net cancer survival is important when comparing social groups with different background mortality rates, as different methods lead to different results.^51^ In this study, we used relative rather than cause‐specific survival to estimate net cancer survival. Cause‐specific survival methods use cause‐of‐death data to estimate net survival from cancer while censoring deaths from other causes (e.g., Kaplan–Meier, Cox models). However, relative survival is generally preferred over cause‐specific survival by cancer registries to measure net cancer survival because it avoids errors due to misclassification of the cause of death.^12^, ^13^ Death certificates require a subjective assessment of the cause of death, which may be difficult to resolve in the presence of multiple underlying health conditions. We opted not to use cause‐specific survival in part due to differences in the prevalence of comorbidities between different groups,^42^ which could lead to differential misclassification of death by the certifier. While relative survival avoids potential misclassification biases from cause of death certification, it can still be a biased estimate of net survival if the comparator is not a good estimate of expected survival in the absence of cancer. Our results suggest that there may be important differences in the background mortality risk of cancer cases compared with general population life tables, and that consequently relative survival compared with life tables likely underestimates net cancer survival, even when using group‐specific life tables.

In conclusion, we believe our study fills an important data gap by providing representative national‐level estimates of net cancer survival stratified by key individual‐level health equity stratifiers for monitoring health equities. We provide estimates using two methods of calculating net survival, including estimates that are comparable with national statistics. Despite universal healthcare access, differences in cancer survival exist by race, immigration status, household income, and education level in Canada, suggesting the root causes of cancer survival inequalities go beyond differences in healthcare access.

## AUTHOR CONTRIBUTIONS

**Talía Malagón:** Conceptualization; methodology; investigation; validation; formal analysis; supervision; visualization; writing – original draft; writing – review and editing. **Sarah Botting‐Provost:** Formal analysis; writing – review and editing; validation. **Alissa Moore:** Formal analysis; validation; writing – review and editing. **Mariam El‐Zein:** Project administration; writing – review and editing; supervision. **Eduardo L. Franco:** Funding acquisition; writing – review and editing; supervision; project administration.

## FUNDING INFORMATION

This study was funded by a Canadian Institutes of Health Research (CIHR) (grant 179901) to E.L. Franco and a salary award from the Fonds de Recherche du Québec Santé to T. Malagón. The analysis presented in this paper was conducted at the Quebec Interuniversity Centre for Social Statistics (QICSS) which is part of the Canadian Research Data Centre Network (CRDCN); the services and activities provided by the QICSS are made possible by the financial or in‐kind support of the Social Sciences and Humanities Research Council (SSHRC), the Canadian Institutes of Health Research (CIHR), the Canada Foundation for Innovation (CFI), Statistics Canada, the Fonds de recherche du Québec and the Québec universities.

## CONFLICT OF INTEREST STATEMENT

SBP and AM have no conflicts of interest to declare. TM was a board member of the International Papillomavirus Society from 2018 to 2024. ELF reports grants to his institution from the Canadian Institutes of Health Research, the National Institutes of Health, and Merck unrelated to the conduct of the study; and personal fees from Merck. MZ and ELF hold a patent related to the discovery “DNA methylation markers for early detection of cervical cancer”, registered at the Office of Innovation and Partnerships, McGill University, Montréal, Québec, Canada.

## ETHICS STATEMENT

We obtained ethical approval from the McGill University Institutional Review Board for this analysis of secondary data.

## Supporting information


Data S1

Table S1

Table S2

## Data Availability

Restrictions apply to the availability of these data, which were used under license by Statistics Canada for this study. Eligible researchers can apply for data access through the Statistics Canada Research Data Centre program (https://www.statcan.gc.ca/en/microdata/data-centres/access). Program code and Poisson model outputs used for the current analyses are available at the Borealis repository at https://doi.org/10.5683/SP3/STER2V. Further information is available from the corresponding author upon request.
